# Thermally adapted *Escherichia coli* keeps transcriptomic response during temperature upshift exposure

**DOI:** 10.1007/s00253-025-13495-1

**Published:** 2025-05-13

**Authors:** Gilberto Pérez-Morales, Karla V. Martínez-Conde, Luis Caspeta, Enrique Merino, Miguel A. Cevallos, Guillermo Gosset, Alfredo Martinez

**Affiliations:** 1 Department of Cellular Engineering and Biocatalyst, Instituto de Biotecnología, Col. Chamilpa, Universidad Nacional Autónoma de México, Av. Universidad 2001, Cuernavaca, Morelos 62210 Mexico; 2 Department of Molecular Microbiology, Instituto de Biotecnología, Col. Chamilpa, Universidad Nacional Autónoma de México, Av. Universidad 2001, Cuernavaca, Morelos 62210 Mexico; 3 Program of Evolutionary Genomics, Centro de Ciencias Genómicas, Col. Chamilpa, Universidad Nacional Autónoma de México, Av. Universidad 2000, Cuernavaca, Morelos 62210 Mexico

**Keywords:** Thermal adaptive laboratory evolution, Temperature upshift exposure, Thermotolerance, d-Lactic acid, Transcriptomic analysis, *Escherichia coli*

## Abstract

**Abstract:**

The heat shock response is a cellular protection mechanism against sudden temperature upshifts extensively studied in *Escherichia coli*. However, the effects of thermal evolution on this response remain largely unknown. In this study, we investigated the early and late physiological and transcriptional responses to temperature upshift in a thermotolerant strain under continuous culture conditions. Adaptive laboratory evolution was performed on a metabolically engineered *E. coli* strain (JU15), designed for d-lactic acid production, to enable cellular growth and fermentation of glucose at 45 °C in batch cultures. The resulting homofermentative strain, ECL45, successfully adapted to 45 °C in a glucose-mineral medium at pH 7 under non-aerated conditions. The thermal-adapted ECL45 retained the parental strain’s high volumetric productivity and product/substrate yield. Genomic sequencing of ECL45 revealed eight mutations, including one in a non-coding region and six within the coding regions of genes associated with metabolic, transport, and regulatory functions. Transcriptomic analysis comparing the evolved strain with its parental counterpart under early and late temperature upshifts indicated that the adaptation involved a controlled stringent response. This mechanism likely contributes to the strain’s ability to maintain growth capacity at high temperatures.

**Key points:**

• *The temperature upshift response of a thermally adapted strain in continuous culture was studied for the first time.*

• *Genomic analyses revealed the presence of a double point mutation in the spoT gene.*

• *The thermally adapted strain maintained underexpression of the spoT gene at high temperatures.*

• *Supplementation of 0.15 g/L of hydrolyzed protein favored thermal adaptation at 45 °C.*

**Supplementary Information:**

The online version contains supplementary material available at 10.1007/s00253-025-13495-1.

## Introduction

Temperature is a key abiotic factor that determines the survival of organisms in a given environment. To thrive outside their thermal niche, organisms must adapt or face extinction (Haynes 1964). The heat shock response (HSR) is a universal protective mechanism triggered by sudden temperature upshifts. In *Escherichia coli*, the HSR is characterized by the increased synthesis of heat shock proteins (HSP), regulated by the transcription factor σ32, when cells are suddenly shifted from 30 to 42 °C.

Although the HSR in *E. coli* has been extensively characterized under well-controlled conditions, most studies have focused on wild-type strains subjected to a temperature upshift to 42 °C in batch cultures, typically during short and transient stress periods. However, heat shock experiments in batch cultures have limitations, such as inconsistencies in interpreting results (Kim et al. 2020). The response of thermally adapted strains to temperature upshifts under continuous culture conditions remains unexplored. Additionally, whether evolutionary processes alter the response to a temperature upshift remains an open question.

Adaptive laboratory evolution (ALE) is a powerful tool for generating adapted strains under controlled selective pressure conditions over time (Portnoy et al. 2011). Various ALE studies have demonstrated the ability of wild-type *E. coli* strains to adapt to high temperatures both in minimal media (Bennett et al. 1990; Kishimoto et al. 2010; Tenaillon et al. 2012; Sandberg et al. 2014a) and in rich media (Rudolph et al. 2010; Blaby et al. 2012; Luan et al. 2015). Recently, we generated thermotolerant strains of a metabolically engineered *E. coli* strain for d-lactic acid production by a thermal ALE under non-aerated conditions (Pérez-Morales et al. 2024). Studying the thermal evolution of genetically modified strains can offer insights into possible differences in adaptive mechanisms between wild-type and genetically modified strains, thus facilitating the rational design of thermotolerant strains for industrial application.

In this study, we evaluated the early and late responses to temperature upshifts in a thermotolerant strain. Thermal ALE via serial passages was used to develop the *E. coli* strain ECL45, capable of growth and fermenting glucose to lactic acid at 45 °C. Kinetic and stoichiometric parameters were assessed at various temperatures to explore physiological changes during thermal adaptation. Comparative genomic analysis identified mutations acquired during the evolutionary process. Furthermore, physiological and transcriptomic analyses in continuous culture provided insights into the adaptive mechanisms underlying the response to temperature upshifts in the thermotolerant strain.

## Material and methods

### Organisms and culture conditions

The *E. coli* JU15 strain (MG1655: Δ*pflB*, Δ*frdA*, Δ*adhA*, Δ*xylFGH*, Δreg 27.3 kpb, *gatC*^S184L^) (Utrilla et al. 2009, 2012, 2016) was used for the thermal adaptive laboratory evolution (TALE) experiments. Parental, intermediate, and endpoint populations and strains were cultivated in AM1 mineral medium containing 0.1 g/L of sodium citrate and 40 g/L of glucose. The composition of AM1 mineral medium (Martinez et al. 2007) was 2.63 g/L (NH_4_)_2_HPO_4_, 0.87 g/L NH_4_H_2_PO_4_, 1.5 mM MgSO_4_·7H_2_O, 1.5 mL/L trace metal solution, 2.0 mM KCl, and 1.0 mM betaine. The trace element solution contains per liter: 1.6 g FeCl_3_, 0.2 g CoCl_2_·6H_2_O, 0.1 g CuCl_2_, 0.2 g ZnCl_2_·4H_2_O, 0.2 g Na_2_MoO_4_, 0.05 g H_3_BO_3_, and 0.33 g MnCl_2_·4H_2_O. The medium was supplemented with 0.1 g/L of tryptone and 0.05 g/L of yeast extract as needed.

### Thermal adaptive laboratory evolution

TALE experiments were conducted in serial batch passages at mid-exponential phase in 300 mL mini-fermenters with 200 mL working volume at 150 rpm, pH 7, and without aeration (Pérez-Morales et al. 2024). The initial inoculum of 0.1 OD_600_ was taken from a liquid culture of the largest colony observed on a Petri dish. Temperature was gradually increased by 0.5–2 °C once the growth rate approached that of the parental strain. The temperature was controlled using a thermocirculator, and pH was maintained constant via the automatically adding 2 N KOH. During the TALE, the growth rate of the population was determined from the mid-exponential growth phase monitored by following the optical density at 600 nm.

The AM1 medium with 40 g/L glucose and 0.1 g/L sodium citrate was used as the base culture medium. At 45 °C, the medium was supplemented with 0.1 g/L of tryptone and 0.05 g/L of yeast extract. Before each temperature increment, 0.8 mL of culture was sampled, mixed with 40% glycerol, and stored at − 70 °C. Monoclonal thermotolerant strains were isolated by selecting and evaluating colonies from cryovial-preserved samples. Finally, six colonies were isolated by the streak plate procedure from the last TALE sample at 45 °C, and the endpoint ECL45 monoclonal strain was selected from the highest growth rate obtained from the cultivation at 45 °C in mini-fermenters containing the glucose minimal medium with 0.1 g/L tryptone and 0.05 g/L yeast extract.

### Physiological analysis

Batch cultures for physiological measurements were performed in 300 mL mini-fermenters containing 200 mL of AM1 medium supplemented with 40 g/L glucose and 0.1 g/L sodium citrate (Beall et al. 1991). Cultures were agitated at 150 rpm without aeration and inoculated at an initial OD_600_ of 0.1. Temperature was controlled using a thermocirculator, and pH was maintained by the automatic addition of a 2 N KOH solution. The medium was supplemented with 0.1 g/L tryptone and 0.05 g/L yeast extract. Kinetic and stoichiometric parameters were determined, like the specific growth rate (*µ*_exp_), the specific yield of D-LAC on glucose (*Y*_p/s_), the specific biomass yield on consumed glucose (*Y*_x/s_), the specific glucose consumption rate (*q*_s_), and the specific D-LAC production rate (*q*_p_) which were derived from growth, residual glucose, and produced d-lactic acid curves obtained during the exponential growth phase. *X*_24_ and *Q*_P24_ represent the biomass and the volumetric productivity of D-LAC at 24 h of cultivation, respectively.

### Evaluation of nutritional supplementation

The effect of nutrient supplementation on the culture medium was evaluated in batch cultures with AM1 minimal medium at 45 °C (from the population in evolution at 43 °C) in 300 mL mini-fermenters as described in the previous section. The concentrations of methionine at 0.075 g/L, casamino acids at 0.045 g/L, and protein hydrolysate at 0.15 g/L were chosen considering a minimal use for *E. coli* growth under non-aerated conditions.

### Chemostat culture conditions

Continuous cultures were performed using the thermotolerant strain ECL45 and the parental strain JU15 as reference in a 1-L bioreactor (Applikon ADI 1010—ez-Control, Delft, NL). For this purpose, the seed inocula were obtained from mid-exponential phase batch cultures in AM1 medium as described above. The bioreactor was operated with 750 mL of working volume at 400 rpm, pH 7 maintained by the automatic addition of 8 N KOH, and without aeration. Batch startup at 37 °C began with an OD_600_ of 0.1 (0.037 g-_DCW_/L). After reaching the stationary phase, the chemostat culture operation was set at a dilution rate (*D*) of 0.09 h^−1^. For temperature upshift experiments, cultures were transitioned to 45 °C after reaching the steady state at 37 °C. The feeding solution contained AM1 medium with 40 g/L of glucose, 0.1 g/L of sodium citrate, 0.1 g/L of tryptone, and 0.05 g/L of yeast extract.

### Genomic analysis

Genomic DNA was extracted from liquid cultures in Luria broth (LB: 10 g/L of tryptone, 5 g/L of yeast extract, and 5 g/L of NaCl) using a DNA extraction kit. Sequencing was performed on the Illumina® NextSeq 500 platform from 2 × 75 bp reads. Genome assembly was performed using BWA-MEM (Li and Durbin 2009) and aligning paired reads to the *E. coli* K12 MG1655 reference genome (NCBI access: NC_000913.3). FastQC (Andrews et al. 2015) and Cutadapt (Martin 2011) programs were used for quality control. SAM/BAM files were manipulated using SAMtools (Li et al. 2009). Genomic annotation was performed with the Prokka program (Seemann 2014) and using the *E. coli* K12 MG1655 reference genome. Mutational analyses of the thermotolerant variants were carried out using the breseq pipeline (Deatherage and Barrick 2014) using the *E. coli* JU15 genome sequence as a reference. The sequencing resulted in a total of 15,546,103 mappable paired-end reads, achieving a read depth of 251×.

### Bioinformatic protein and domain structure analysis

The effects of coding gene mutations were evaluated at the protein structure level to determine possible alterations and damage in protein structure and Pfam domains. Amino acid substitutions were simulated using the Missense3D (Ittisoponpisan et al. 2019). Protein 3D structures were retrieved from the UniProt database as a PDB and AlphaFold files. Pfam domains were analyzed using the Pfam database provided by InterPro (Blum et al. 2025).

### Transcriptomic analysis

Microarray samples of parental JU15 and evolved strain ECL45 were collected during the chemostat culture at (a) 37 °C steady state, (b) 45 °C after 15 min of heat shock, and (c) 45 °C after three residence times. Samples were stored in an RNA degradation inhibitory solution at − 70 °C. RNA was extracted using the hot phenol method (Flores et al. 2005) and treated with TURBO DNA-free™. RNA quality was evaluated in a 1% agarose gel electrophoresis, and concentrations were determined using the nanodrop spectrophotometric method. The cDNA synthesis and microarray hybridization were performed by the DNA Microarrays Unit of the Institute of Cellular Physiology at the Universidad Nacional Autónoma de México. For probe preparation and hybridization to arrays, 10 µg of total RNA was used for cDNA synthesis, incorporating dUTP-Alexa555 or dUTP-Alexa647, using the SuperScript Plus direct labeling kit (Invitrogen). The incorporation of the fluorophore was analyzed by measuring the absorbance at 555 nm and 647 nm, respectively. Equal quantities of labeled cDNA were hybridized with the hybridization solution HybIT2 (TeleChem International, Inc.). The arrays were incubated for 14 h at 42 °C and then washed three times with 1X SCC and 0.05% SDS at room temperature. Acquisition and quantification of array images were performed using a GenePix 4100 A reader along with its accompanying software. Differential expression analysis was performed with *genArise* (Gomez-Mayen et al. 2019)*,* using a *Z*-score cutoff of |*Z*|> 1.5. The raw data from microarrays was deposited in the GEO DataSets with accession numbers GSE289056 in the NCBI (token sdyhmuqkxzsxtkx https://www.ncbi.nlm.nih.gov/geo/query/acc.cgi?acc=GSE289056). The enrichment analysis of Gene Ontology (GO) terms and KEGG pathways was performed using the *clusterProfiler* library in R software with a *p*_adj_-value < 0.01 and *q*-value < 0.05 (Wu et al. 2021). Transcription factors (TFs) were retrieved from the RegulonDB database (Tierrafría et al. 2022) using the *regutools* library in R software (Chávez et al. 2020). The independently modulated gene sets (iModulons) were retrieved from iModulonDB (Rychel et al. 2021). Heatmaps and Venn diagrams were plotted using the *pheatmap* libraries (Kolde 2022) and *VennDiagram* (Chen 2022) libraries, respectively.

### Analytical methods

Cell concentration was determined indirectly by the optical density at 600 nm using a GENESYS 10S UV–Vis spectrophotometer (Thermo Scientific). The OD_600_ was converted to dry cell weight (DCW) per liter using the relationship 1 OD_600_ = 0.37 g_-DCW_/L. Supernatants were stored at − 4 °C for glucose and d-lactic acid quantification by high-performance liquid chromatography (HPLC) (Waters, MA, USA). The analysis was performed using an Aminex HPX-87H column (Bio-Rad, Hercules, CA) operated at 60 °C. The mobile phase consisted of a 5 mM H_2_SO_4_ solution at 0.5 mL/min flow rate. Metabolites were detected using a diode array detector (Waters 996, MA, USA) and a refractive index detector (Waters 410, MA, USA). The concentration of glucose and d-lactic acid was calculated from calibration curves obtained with pure standards.

## Results

### Minimal supplementation with hydrolyzed protein enabled the development of a thermotolerant *E. coli* JU15 in the glucose-mineral medium through batch serial transfers

**Fig. 1:**
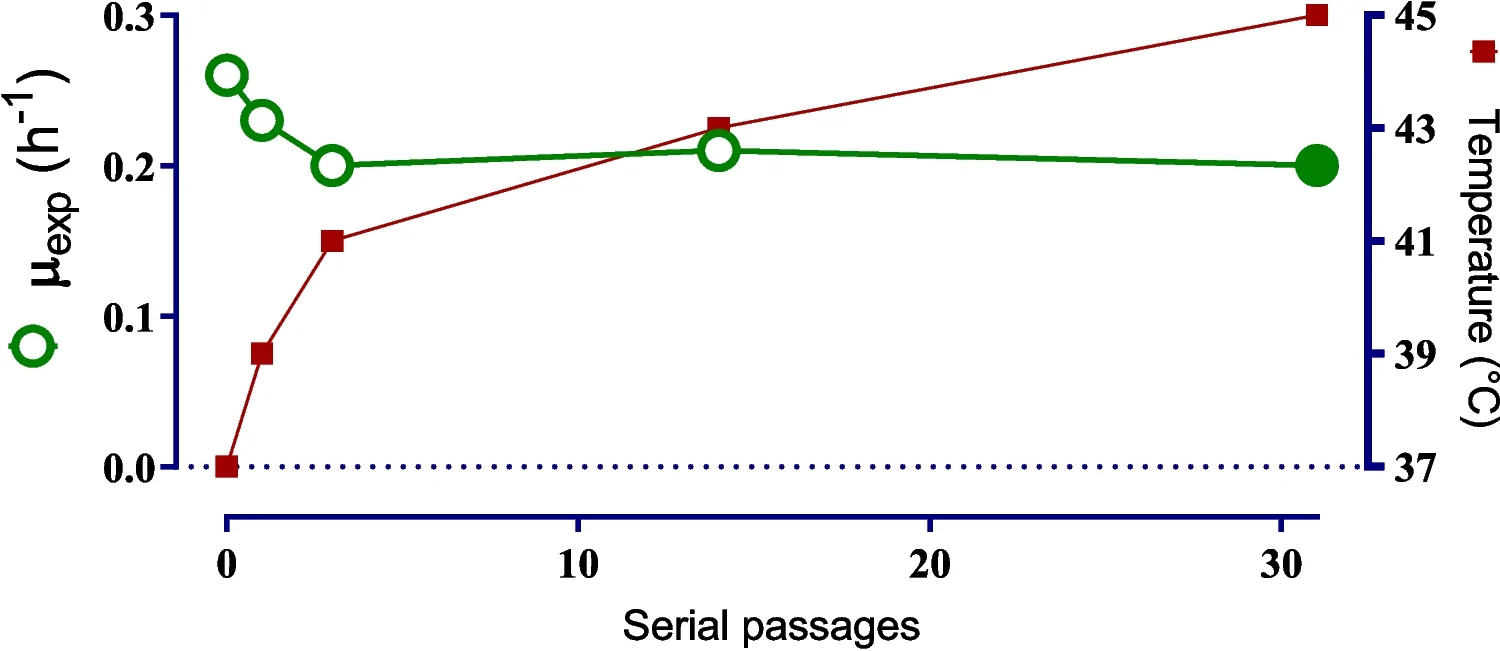
Fitness growth during batch serial transfers of the TALE of the thermally adapted cell population derived from *E. coli* JU15. The data at 37 °C represents the parental strain JU15, while values from 39 to 45 °C correspond to evolved thermally adapted populations. The filled circle represents the endpoint ECL45 strain with 0.15 g/L of hydrolyzed protein supplementation

The homofermentative strain *E. coli* JU15 was subjected to a TALE process under non-aerated conditions in the glucose-mineral medium at pH 7, with temperatures gradually increased above 37 °C. The TALE experiment involved batch serial transfers using the homofermentative *E. coli* strain JU15 and incrementally raising the temperature from 37 to 45 °C, until the population regained its growth rate (Fig. 1). Initially, temperature increments of 2 °C were applied. At 41 °C, the evolving population maintained a specific growth rate (*µ*) above 0.20 h^−1^, compared to a *µ* of 0.26 h^−1^ observed in the parental JU15 strain at 37 °C. However, further temperature increases beyond 41 °C significantly impacted the growth rate of the population. To allow adaptation to proceed, smaller temperature increments of 0.5 °C were applied, accompanied by multiple serial transfers. Between 41 and 43 °C, 11 serial transfers were required to improve the growth rate from 0.11 h^−1^ to approximately 0.20 h^−1^ (data not shown).

**Fig. 2:**
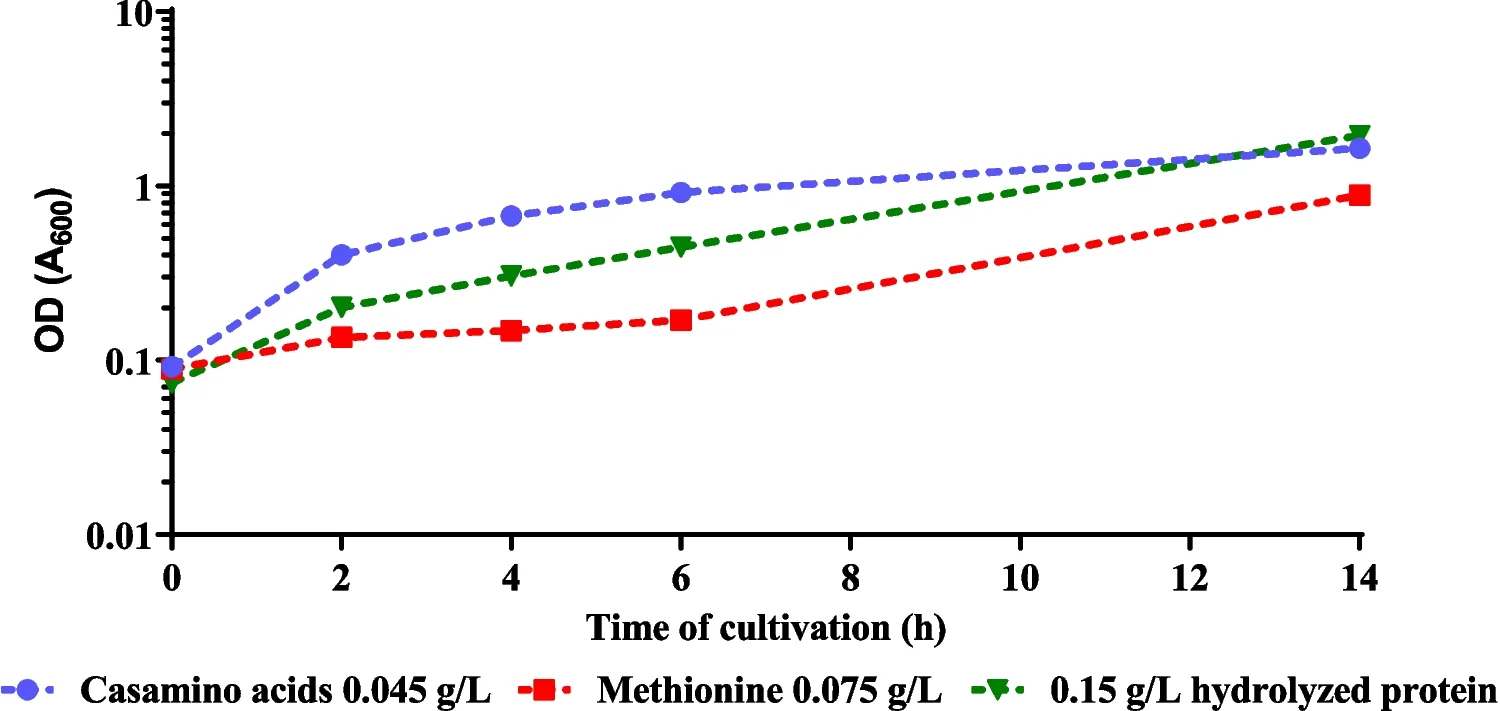
Effect of supplementation components on growth at 45 °C. The thermally adapted population at 43 °C was evaluated in AM1 minimal medium supplemented with casamino acids (filled circles), methionine (filled squares), and hydrolyzed protein (filled triangles)

At temperatures above 43 °C, the population failed to grow even with incremental increases of 0.5 °C. Previous studies have demonstrated that the limitation in thermotolerance at temperatures above 42 °C is associated with the loss of the ability to synthesize methionine and other cofactors (Chang et al. 2013; Mordukhova and Pan 2013). To overcome this limitation, supplementation with compounds related to methionine metabolism was evaluated to mitigate biosynthetic constraints at high temperatures. As shown in Fig. 2, supplementation with methionine, casein hydrolysates (casamino acids), and hydrolyzed protein from LB medium restored cell growth. While casamino acids had a greater positive effect on growth compared to methionine and LB components, they did not support the selection of viable colonies. Consequently, supplementation with 0.15 g/L of hydrolyzed protein (comprising 0.1 g/L tryptone and 0.05 g/L yeast extract, equivalent to 1% of the hydrolyzed protein contained in the LB medium) was implemented. With this supplementation, and after 17 additional serial transfers, the growth rate increased to 0.2 h^−1^ at 45 °C (Fig. 1). In total, after 31 serial transfers and 126 generations, a thermotolerant strain, derived from JU15 and capable of growing at 45 °C and pH 7, under non-aerated conditions in glucose-mineral medium supplemented with 0.15 g/L of hydrolyzed protein, was obtained. The monoclonal strain exhibiting the highest growth rate among the six isolated colonies was designated ECL45 (Supplementary Fig. S2).

### The thermally adapted strain maintains kinetic and stoichiometric parameter values at elevated temperatures

**Fig. 3:**
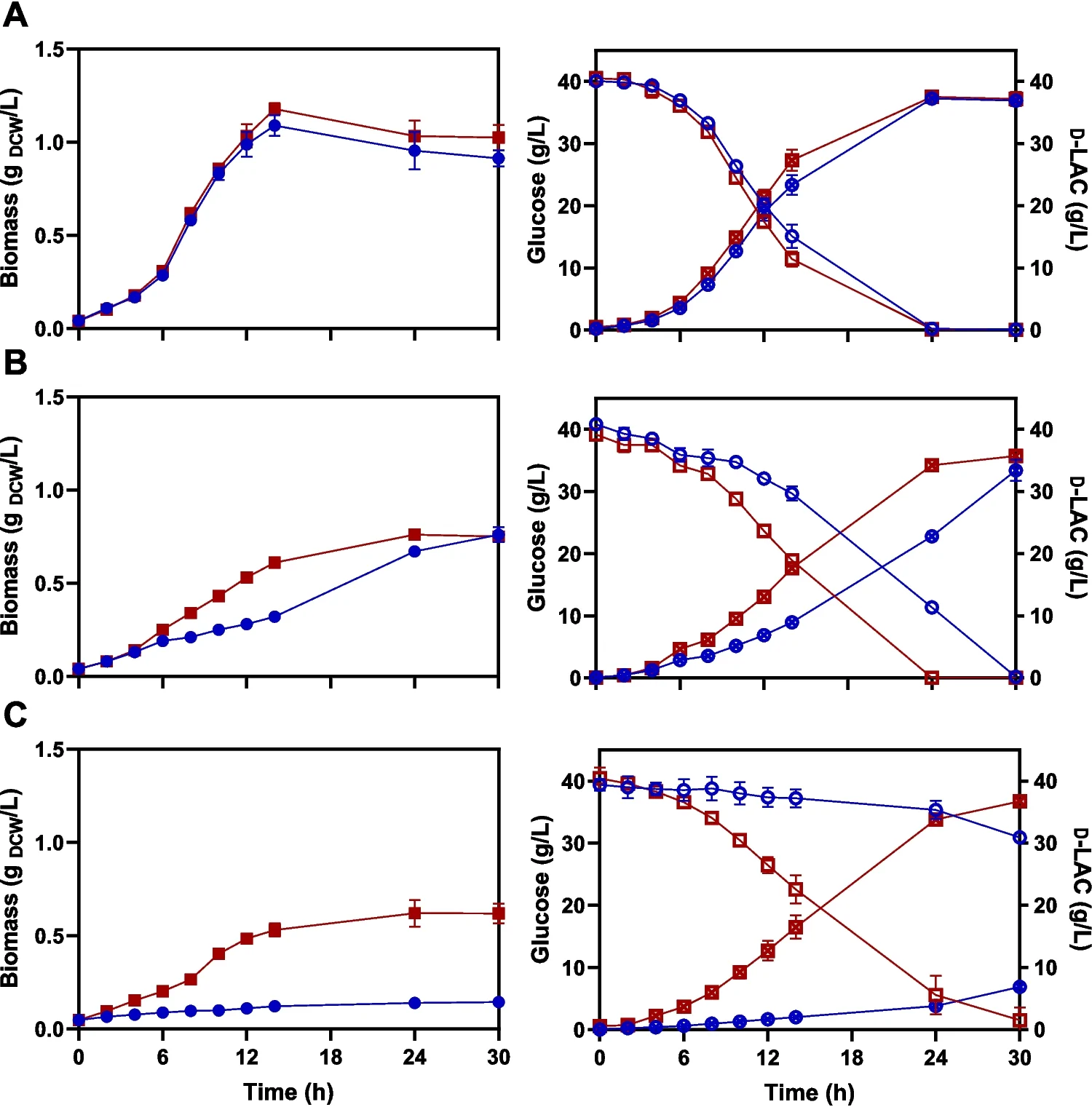
Fermentation kinetics of parental (JU15) and thermally adapted (ECL45) strains in glucose-mineral medium supplemented with 0.15 g/L hydrolyzed protein at various growth temperatures (**A** 37 °C, **B** 43 °C, and **C** 45 °C). Dark blue points represent JU15 kinetics, while dark red points correspond to strain ECL45

The TALE experiment generates a diverse population of thermally adapted strains. Six colonies were isolated and evaluated in batch fermentations at different growth temperatures to identify a monoclonal strain with superior performance. The monoclonal strain with the highest specific growth rate (*µ*) was selected for detailed assessment across various temperatures in batch cultures. The kinetic and stoichiometric parameters of the parental JU15 strain and the thermally adapted ECL45 strain were determined using AM1 mineral medium supplemented with 40 g/L of glucose and 0.15 g/L of hydrolyzed protein at pH 7 and non-aerated conditions (Table 1). As shown in Fig. 3, an increase in culture temperature significantly reduces the growth performance of JU15. In contrast, the thermally adapted strain ECL45 demonstrated robust growth and effective glucose conversion to d-lactic acid at temperatures up to 45 °C. At 37 °C, both strains showed similar kinetic and stoichiometric parameters. At 43 °C, while both strains completed the glucose to d-lactic acid conversion, ECL45 outperformed JU15 in fermentation metrics, showing higher specific rates of glucose consumption and d-lactic acid production. Interestingly, at 45 °C, ECL45 exhibited a 47% reduction in growth rate compared to 37 °C but maintained high volumetric productivity and product/substrate yield (Table 1). In contrast, JU15 failed to grow or metabolize glucose efficiently within the 30-h fermentation period evaluated at this temperature.

**Table 1:** Kinetic and stoichiometric parameters of parental and evolved strains in glucose-mineral medium with 0.15 g/L hydrolyzed protein. ND, not determined

Parameter	Strain	Growth temperature (°C)		
		37	43	45
*µ*_exp_ (h^−1^)	JU15	0.29 ± 0.01	0.07 ± 0.00	ND
	ECL45	0.30 ± 0.00	0.32 ± 0.01	0.16 ± 0.01
*Y*_p/s_ (g_D-LA_/g_Glc_)	JU15	91.32% ± 0.47	81.21% ± 4.77	ND
	ECL45	90.20% ± 2.58	91.71% ± 1.45	91.22% ± 2.05
*Y*_x/s_ (g_DCW_/g_Glc_)	JU15	5.77% ± 0.04	1.96% ± 0.14	ND
	ECL45	5.12% ± 0.29	4.36% ± 0.50	2.99% ± 0.24
*q*_s_ (g_Glc_/g_DCW_ h)	JU15	5.08 ± 0.10	3.66 ± 0.29	ND
	ECL45	5.92 ± 0.41	7.28 ± 0.71	5.44 ± 0.80
*q*_p_ (g_D-LA_/g_DCW_ h)	JU15	4.64 ± 0.12	2.96 ± 0.06	ND
	ECL45	5.35 ± 0.52	6.67 ± 0.55	4.95 ± 0.62
*Q*_P24_ (g/L h)	JU15	1.55 ± 0.02	0.95 ± 0.02	ND
	ECL45	1.56 ± 0.00	1.43 ± 0.00	1.41 ± 0.04
*X*_24 h_ (g_DCW_/L)	JU15	0.95 ± 0.10	0.67 ± 0.00	ND
	ECL45	1.03 ± 0.08	0.76 ± 0.00	0.62 ± 0.07

### Genomic mutations affect some protein structures during thermal adaptation

One aim of the ALE experiments was to allow the identification of genomic changes resulting from the adaptation process. Accordingly, the genome of the thermally adapted strain ECL45 (evolved to grow at 45 °C) was sequenced. Eight mutations were identified, including six non-synonymous mutations, one intergenic mutation, and one single base-pair deletion (Table 2). These mutations are associated with metabolic processes such as isopentenyl diphosphate biosynthetic process (*dxr*), cysteine biosynthetic process (*cysJ*), the stringent response and starvation adaptation (*spoT*), and arginine biosynthetic process (*argE*). Some mutations are also related to membrane-associated functions, such as the putrescine ABC exporter ATP-binding protein (*sapD*), and transcriptional regulation, such as the DUF179 domain-containing protein (*yqgE*).

**Table 2:** Mutational changes in thermally adapted strain ECL45

Position	Mutation	Annotation	Gene	Description
193,995	A → C	S159R (AGT → CGT)	*Dxr → *	1-Deoxy-d-xylulose 5-phosphate reductoisomerase
1,352,925	C → G	G235 A (GGT → GCT)	*sapD ← *	Putrescine ABC exporter ATP-binding protein
1,572,082	(A)_6→5_	Intergenic (−37/+ 325)	*gadB ←/← pqqL*	Glutamate decarboxylase B/periplasmic metalloprotease
2,890,521	G → T	P460 T (CCA → ACA)	*cysJ ← *	Sulfite reductase, flavoprotein subunit
3,093,194	Δ1 bp	Coding (258/564 nt)	*yqgE → *	DUF179 domain-containing protein
3,823,228	C → G	L277 V (CTG → GTG)	*spoT → *	Bifunctional (p)ppGpp synthase/hydrolase
3,823,766	C → T	A456 V (GCT → GTT)	*spoT → *	Bifunctional (p)ppGpp synthase/hydrolase
4,154,104	G → T	D248E (GAC → GAA)	*argE ← *	Acetylornithine deacetylase

**Fig. 4:**
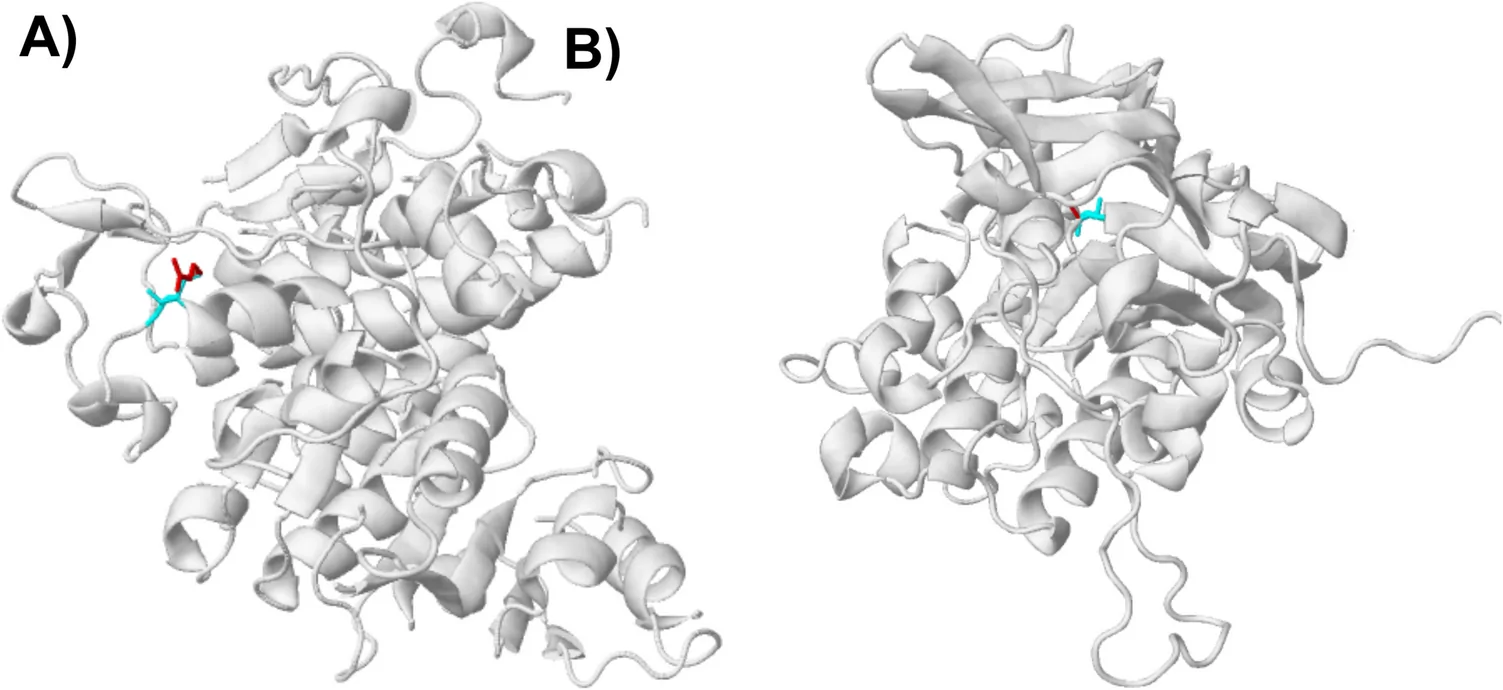
Bioinformatic structural analysis of protein variants. Wild-type (blue) and mutated (red) amino acids are highlighted on the structural model. **A** 3D model of the 1-deoxy-d-xylulose 5 phosphate reductoisomerase (DXR) and **B** the putrescine ABC exporter ATP-binding protein (SAPD)

A single base-pair deletion (G) in *yqgE* altered the protein sequence, substituting leucine for phenylalanine at position 85, and truncated the open reading frame from 187 to 145 amino acids (Supplementary Fig. S1). Bioinformatic structural analysis of the protein variants revealed that only two mutations were predicted to cause structural damage: DXR^S159R^ and SAPD^G235 A^ (Fig. 4). The non-synonymous mutation in *dxr* affected the C-terminal catalytic domain (Pfam: PF08436), resulting in structural damage by shifting the amino acid conformation from a buried state to an exposed state. Similarly, the mutation in the C-terminal region of the *sapD* gene (Pfam: PF08352) replaced a buried glycine residue with an alanine residue. The amino acid substitutions in CYSJ^P460 T^ (associated with the FAD-binding domain, Pfam: PF00667), ARGE^D248E^ (peptidase dimerization domain, Pfam: PF07687), and SPOT^L277 V, A456 V^ (Pfam: PF04607 and PF19296, respectively) were predicted not to cause structural damage.

### Transcriptomic response at the early and late temperature upshift exposure in continuous culture

**Fig. 5:**
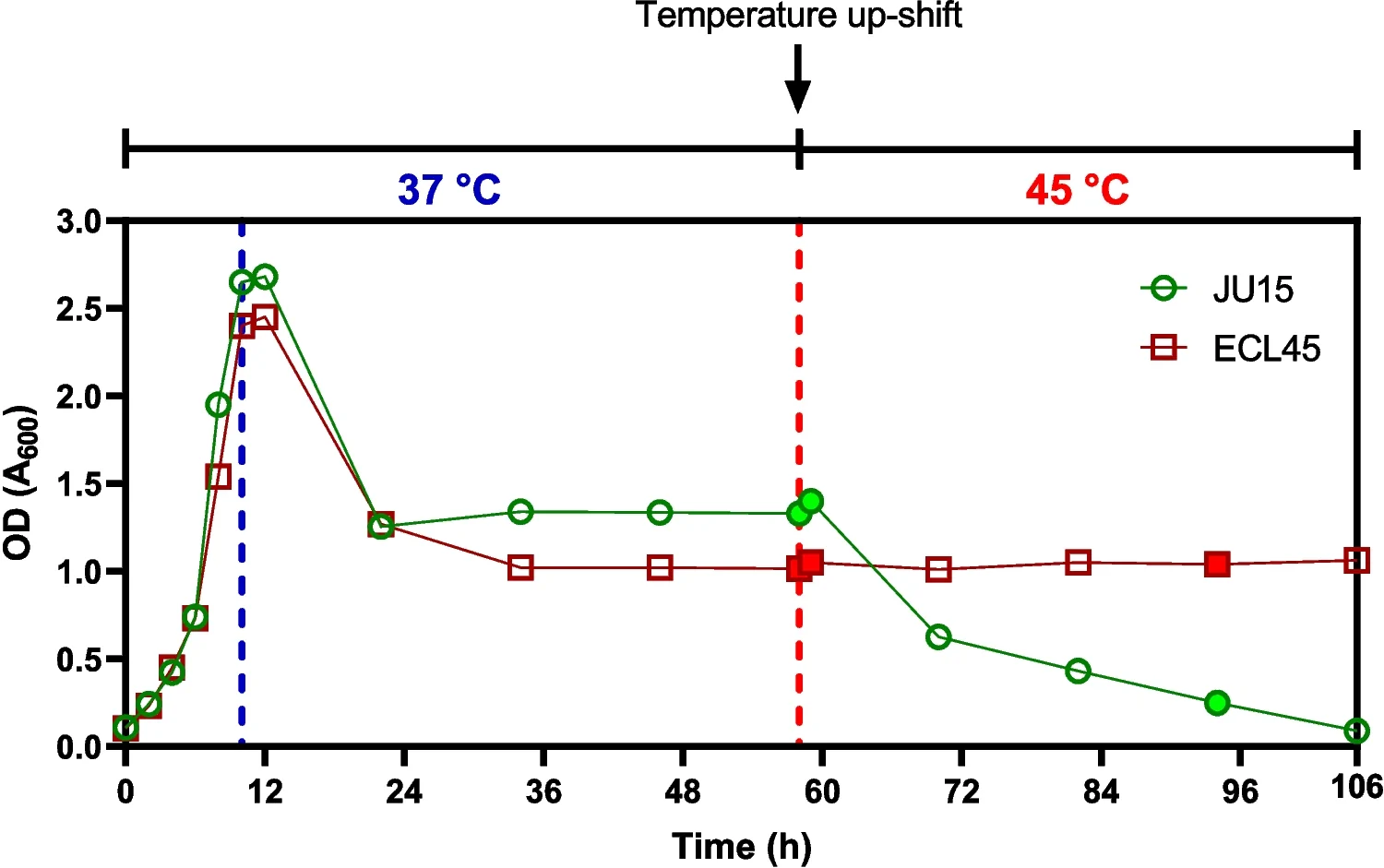
Temperature upshift exposure in steady-state chemostat culture of parental JU15 and thermotolerant ECL45 strain. Filled points represent the sampling points for transcriptomic analysis

**Fig. 6:**
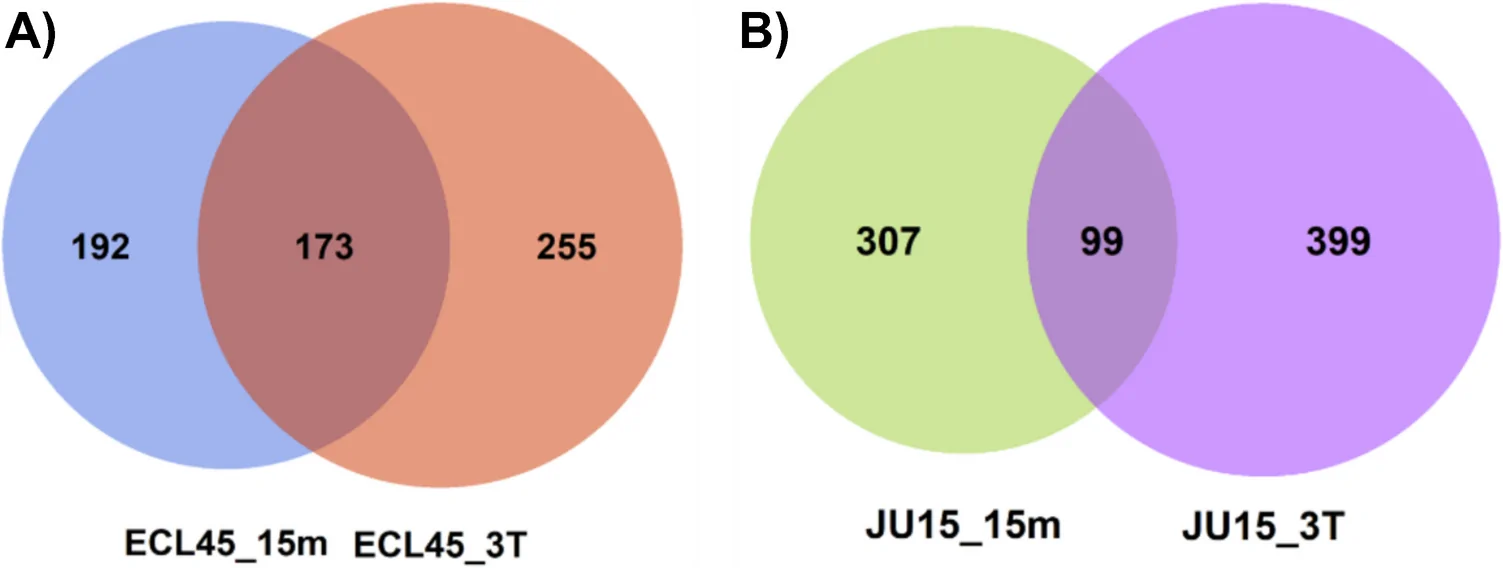
Venn diagrams of differentially expressed genes during early (ECL45_15m and JU15_15m) and late (ECL45_3T and JU15_3T) temperature upshift exposure of parental and thermally adapted strains. **A** Venn diagram of thermotolerant ECL45 strain; **B** Venn diagram of parental JU15 strain

Transcriptomic analyses of thermally adapted strains have typically been previously conducted under batch and transient short-term heat shock conditions. However, the response of a thermally adapted strain to early and late temperature upshift exposure has not been explored. To address this issue, the analysis of differentially expressed genes (DEGs) was performed using RNA microarrays of the thermally adapted strain (ECL45) and the parental strain (JU15) in continuous cultures. RNA samples were collected at the early (15 min) and late (three residence times after the temperature increase to 45 °C) phases of the temperature upshift (Fig. 5). Comparisons were made using the transcriptome at 37 °C, the ancestral optimal growth temperature, as the reference state. The temperature of 45 °C was selected considering the maximum temperature at which the evolved strain ECL45 can sustain growth in batch cultivation. A *Z*-score threshold greater than 1.5 and a *p*-value < 0.05 were used to identify DEGs. The Venn diagram illustrates DEGs identified at the early and late phases of the response (Fig. 6). The thermally adapted strain ECL45 displayed 365 and 428 DEGs in the early and late responses, respectively, while JU15 exhibited a higher transcriptomic response with 406 and 498 DEGs in these phases. Interestingly, ECL45 demonstrated nearly twice as many shared DEGs between the early and late phases compared to JU15. All DEGs were classified into second-level Gene Ontology (GO) categories, including “biological process,” “cellular component,” and “molecular function”. In the “biological processes” GO term category, the predominant GO terms across all analyses, appearing in both up- and downregulated DEGs, included “cellular process,” “metabolic process,” “response to stimulus,” “biological regulation,” and “localization” (Supplementary Table S1).

**Fig. 7:**
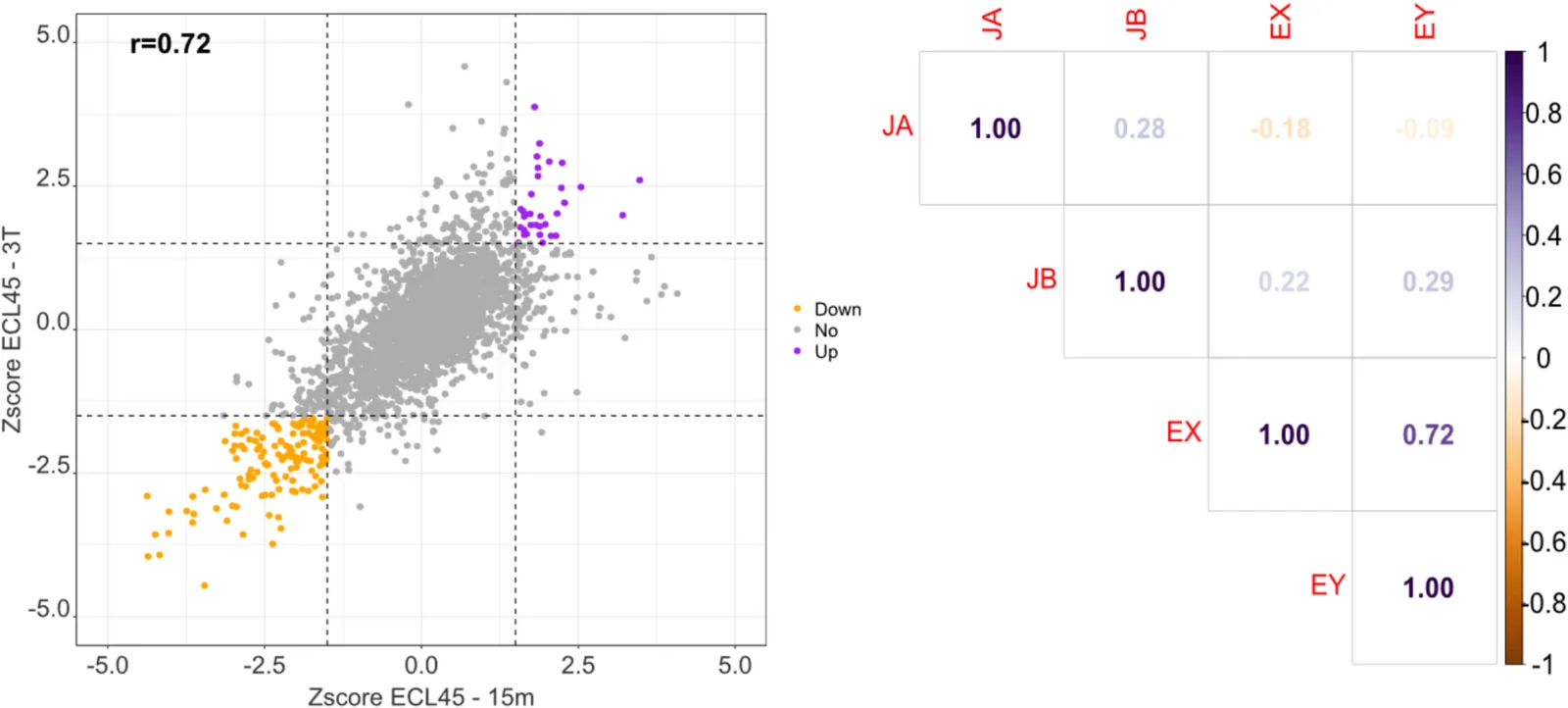
Correlation analysis of heat shock transcriptomic responses in parental JU15 and thermotolerant ECL45 strains. Left half: Scatterplot of ECL45 strain comparing early and late temperature upshift responses. Right half: Correlation matrix between all pairs of microarray comparisons. Microarray experiments are represented as follows: JA, JU15 strain at 45 °C for 15 min compared to 37 °C; JB, JU15 strain at 45 °C for three residence times compared to 37 °C; EX, ECL45 strain at 45 °C for 15 min compared to 37 °C; EY, ECL45 strain at 45 °C for three residence times compared to 37 °C

**Fig. 8:**
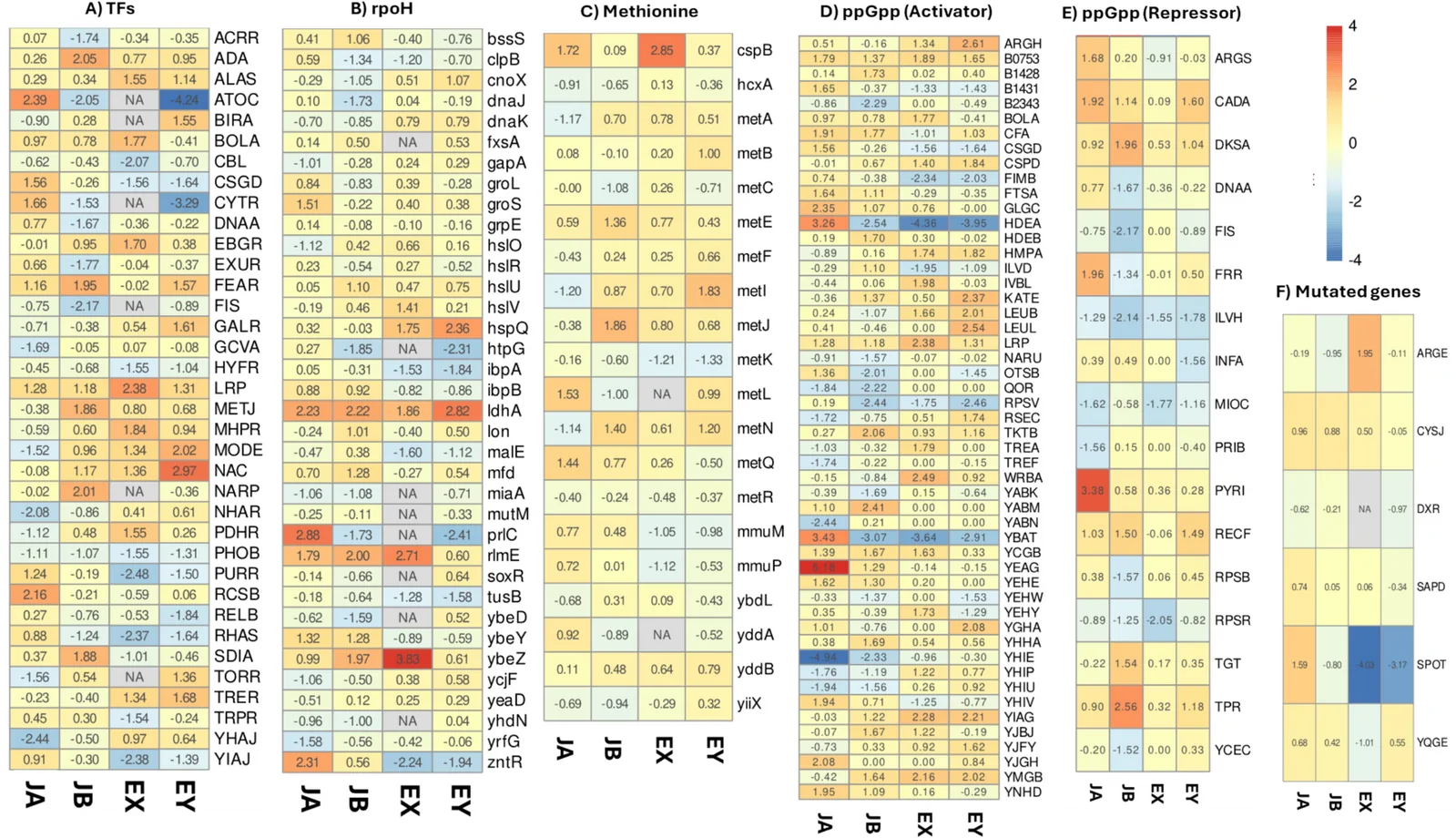
Heatmaps of differentially expressed gene (DEG) clusters during temperature upshift exposure. **A** Transcription factors, **B** rpoH iModulon, **C** methionine iModulon, **D** genes activated by ppGpp, **E** genes repressed by ppGpp, and **F** mutated genes. Microarray experiments are represented as follows: JA, JU15 strain at 45 °C for 15 min compared to 37 °C; JB, JU15 strain at 45 °C for three residence times compared to 37 °C; EX, ECL45 strain at 45 °C for 15 min compared to 37 °C; EY, ECL45 strain at 45 °C for two residence times compared to 37 °C

Compared to the significant reduction in biomass production observed in the parental strain JU15 during temperature upshift exposure, the thermotolerant ECL45 maintained its growth capacity throughout the cultivation period (Fig. 5). Correlation analysis of global transcriptomic patterns across sample pairs revealed that only ECL45 exhibited a strong correlation coefficient (*r* = 0.72, *p-*value < 0.05) between early and late responses (Fig. 7). As shown in Fig. 7, non-DEGs, upregulated, and downregulated genes displayed proportional increases in the *Z*-score values across both states. While the DEGs across all samples did not show significant GO or KEGG enrichment, the analysis of TFs and iModulons groups identified specific genes with altered expression in some microarray comparisons (Fig. 8). A total of 36 TFs were differentially expressed in at least one comparison. Notably, *lrp* but not *csgD* was consistently upregulated, while *phoB* but not *rhaS* was consistently downregulated in ECL45 during both early and late temperature upshift responses.

The methionine iModulon heatmap highlighted *metJ* regulator as the only upregulated DEG during the late temperature upshift in JU15. The RpoH iModulon, representing heat shock–related genes, exhibited distinct expression changes between early and late temperature upshifts in both parental and evolved strains. Interestingly, the *ldhA* gene, which participates in lactic acid production, was consistently upregulated across all phases of the temperature upshift. Other genes with consistent expression patterns in ECL45 included the upregulation of *hspQ* genes and the downregulation of *ibpA* and *zntR*. According to Fig. 8, the mutated *spoT* gene exhibited remarkable differential expression in ECL45 during both early and late temperature upshifts. Further analysis of genes regulated by the ppGpp alarmone revealed proportional expression changes relative to *spoT* in JU15. However, the upregulation of the *dksA*, another gene associated with *ppGpp* regulation, was observed exclusively in JU15 during the late response.

## Discussion

### Genetic background affects the fitness growth adaptation to high temperatures

In recent years, various strategies have been implemented to enhance the thermotolerance of *E. coli*. However, many approaches only provide short-term thermotolerance, limiting their application in long-term high-temperature bioprocesses. The efficacy of TALE experiments for obtaining thermotolerant *E. coli* strains has been well-documented (Kishimoto et al. 2010; Rudolph et al. 2010; Blaby et al. 2012; Tenaillon et al. 2012; Sandberg et al. 2014; Luan et al. 2015). These experiments, however, have predominantly been conducted on wild-type strains with unaltered metabolism and reducing power capacity. In this study, a TALE experiment was used to enhance the thermotolerance of a metabolically engineered *E. coli* strain designed for lactic acid production. This strain was cultivated under non-aerated conditions in a minimal medium supplemented with glucose at pH 7. During the TALE process, serial batch transfers supplemented with components of the LB-rich medium, tryptone, and yeast extract were employed. While wild-type *E. coli* has been reported to adapt to temperatures of 44.8 °C in the M9 minimal medium (Kishimoto et al. 2010), the engineered strain JU15 could only adapt up to 43 °C in the AM1 mineral medium without rich media supplementation. This limitation likely stems from the JU15 strain’s genetic background, which compromises its reducing power to produce two molecules of lactate from one molecule of glucose, affecting biomass generation. Moreover, most ALE studies using wild-type *E. coli* strains suggest that thermal adaptation to 42 °C can be achieved with minimal difficulty (Bennett et al. 1990; Tenaillon et al. 2012; Sandberg et al. 2014a).

At temperatures above 42 °C, various thermosensitive, growth-limiting proteins, primarily involved in the biosynthesis of cofactors and amino acids, become critical. Supplementation with biotin, pantothenate, and methionine has been shown to alleviate these growth limitations (Chang et al. 2013; Mordukhova and Pan 2013). In this study, the supplementation of 1% hydrolyzed protein (i.e., 0.15 g/L) present in the LB-rich medium enabled the lactic acid producer strain to evolve up to 45 °C in batch serial transfers. Previous TALE experiments with LB-rich conditions have allowed wild-type *E. coli* to adapt to temperatures up to 48 °C (Rudolph et al. 2010; Blaby et al. 2012), but such conditions may not be economically feasible for industrial application. Here, minimal supplementation (0.1 g/L tryptone and 0.05 g/L yeast extract) was sufficient to enable the strain to adapt to temperatures up to 45 °C during serial batch transfers.

### The *spoT *gene-mediated stringent response is a common target during thermal adaptation

Mutations acquired during thermal adaptation are often specific to the experimental conditions of the TALE process (Sandberg et al. 2014). Here, we compare our findings with a previous TALE experiment conducted at temperatures exceeding 42 °C in the mineral medium under non-aerated conditions. In this sense, Kishimoto et al. (2010) reported a total of 8, 15, and 21 mutations in the variants isolated at 41.2 °C (41B), 43.2 °C (43B), and 44.8 °C (45 A), respectively. Non-synonymous mutations accumulated over extended generations at the higher temperature of 45 °C. In this study, eight mutations were identified in the endpoint strain ECL45, comparable to the 41B strain from Kishimoto et al. (2010) but significantly fewer than the 21 mutations in their 45 A strain. Interestingly, the *spoT* gene was the only common mutated gene between this study and Kishimoto et al.’s TALE experiments. While the 43B strain had a premature stop codon in the *spoT* gene, ECL45 exhibited two non-synonymous point mutations that do not cause structural damage, according to structure prediction analysis. The repeated occurrence of *spoT* mutations across independent TALE experiments (Blaby et al. 2012; Luan et al. 2015) suggests convergent evolution at the gene level in both mineral and rich media.

### The evolved *spoT*-mediated stringent response maintains transcriptomic homeostasis during temperature upshift

Transcriptomic analyses of thermally adapted strains have previously indicated that heat shock response patterns are not significantly altered during thermal evolution (Ying et al. 2015). However, those studies primarily focused on short-term, transient stress in batch cultures. In this study, continuous cultures were used to evaluate transcriptomic responses to early and late temperature upshifts. The thermally adapted strain exhibited consistent transcriptomic patterns between early and late responses, suggesting that its adaptive mechanism for early transcriptomic reorganization during temperature upshifts is sustained over prolonged exposure. This contrasts with the parental strain JU15 which could only sustain growth during the early response, highlighting its inability to maintain a robust heat shock response over time. Furthermore, the lack of correlation in the early response between the evolved strain ECL54 and the parental strain JU15 indicates that the transcriptomic organization of the heat shock response is altered during thermal evolution.

The results suggest that the thermally adapted strain utilizes a mechanism involving *spoT*-mediated regulation of the magic spot ppGpp to sustain growth at high temperatures. The downregulation of the *spoT* gene in ECL45 during early and late temperature upshifts indicates a controlled stringent response to prevent excessive accumulation of (p)ppGpp, thereby maintaining biosynthetic activity and promoting cell division as an adaptive mechanism (Bremer and Dennis 2008; Spira and Ospino 2020). Conversely, the parental JU15 upregulated *spoT* and *dksA*, leading to increased ppGpp levels and the activation of the stringent response, which hindered growth under high-temperature conditions (Potrykus and Cashel 2008). Unlike a ppGpp^0^ strain (Δ*relA*, Δ*spoT*), ECL45 maintained basal ppGpp levels, enabling balanced growth and responsiveness to environmental stimuli (Potrykus et al. 2011). Considering the regulatory role of ppGpp in transcription (Kaczanowska and Rydén-Aulin 2007), this suggests that *spoT* mutations may restore transcriptome homeostasis, as observed in other studies of regulatory mutations (Rodríguez-Verdugo et al. 2016). In addition to the reduced expression of the *spoT* gene, the amino acid substitution in the protein, associated with the catalytic domain of synthase activity and the intermediate region responsible for allosteric regulation, may be impairing synthase activity and its ability to interact with other proteins in response to stress stimuli (Fujita et al. 2002; Potrykus and Cashel 2008).

### Mutational changes enhance growth capacity and lactic acid production at elevated temperatures

Overall, all positive mutations aim to restore transcriptomic homeostasis. Rodriguez-Verdugo et al. (2016) found that this restoration occurs during the early stages of the thermal adaptation process mediated by mutations in *rpoB*. In our study, no mutation was detected in the RNA polymerase complex. However, the overexpression of *rpoB* in the early response and the reduced expression of *rpoC* in the late response in the parental strain JU15 suggest that the mutations acquired during the adaptation process endowed the thermotolerant strain with the ability to restore transcriptomic homeostasis through a mechanism associated with what was previously discussed regarding *spoT*. *yqgE* is a non-essential gene of unknown function. The disruption of the open reading frame may alter the genetic network involved in DNA repair (Al Mamun et al. 2012). The enzyme 1-deoxy-d-xylulose 5-phosphate reductase (DXR) plays an essential role in the methylerythritol phosphate (MEP) pathway for isoprenoid biosynthesis. Previous studies have shown that mutations at positions H153, H209, and H257 of the DXR protein decrease its affinity for the substrate (Kuzuyama et al. 2000). In this context, the predicted structural damage at position 159 in the DXR protein could affect substrate binding, favoring the flow of carbon towards glycolysis and restricting the MEP pathway. The SapD protein, encoded by the *sapD* gene, belongs to the ABC (ATP-binding cassette) family of transporters that facilitate the ATP-mediated transport of potassium (TrkH/TrkG system) and polyamines (SapBCDF) (Harms et al. 2001; Sugiyama et al. 2016). The predicted structural change may affect the conformation of the ATPase or its interaction with other subunits of the transporter complex, thus promoting the transport of K^ +^ and polyamines to maintain cellular homeostasis.

The effects of the mutations are reflected in the kinetic and stoichiometric parameters. The specific biomass-to-substrate yield suggests that these mutations enable the thermally adapted strain to grow at 45 °C, possibly at the expense of increased maintenance energy. Despite this, the thermally adapted strain can maintain the product-to-substrate yield and volumetric productivity at a lower cell concentration, since it sustains the specific substrate consumption rate and the specific lactic acid production rate similar to fermentation at 37 °C. Furthermore, the overexpression of the *ldhA* gene during the steady state at 45 °C suggests that lactic acid biosynthesis is highly active.

### *ldhA* overexpression may likely alleviate heat shock protein expression during the temperature upshift response in lactic acid–producing *E. coli* strains under non-aerated conditions

HSP are commonly expressed immediately after a temperature upshift and subsequently reach a steady-state. During a temperature increase in the continuous culture of an *E. coli* strain, the relative expression levels of the main heat-shock genes were observed to increase (Hasan and Shimizu 2008; Kim et al. 2020). In this study, a few heat shock genes were differentially expressed in the thermally adapted strain, suggesting that the heat shock response is not the primary response during the temperature upshifts. While JU15 increases *groS* overexpression only during the early response, ECL45 maintains *hspQ* overexpression during the late response. Furthermore, strain JU15 reduces long-term *dnaJ* expression. No heat shock–related transcription factors such as *rpoH*, *rpoE*, or *rpoS* showed differential expression. Surprisingly, in both the parental strain JU15 and the thermoadapted strain, the primary overexpressed gene is *ldhA*. Previous studies have reported up to a 13-fold increase in the relative expression of *ldhA* (Zhao et al. 2005). Furthermore, lactate production is favored under microaerobic conditions due to the upregulation of *ldhA* gene expression at high temperatures in continuous culture (Hasan and Shimizu 2008). These findings allow us to hypothesize that *ldhA* gene up-expression may alleviate HSP expression during the temperature upshift response in both parental JU15 and thermotolerant ECL45 strains.

Overall, this study demonstrates the adaptive process of a metabolically engineered *E. coli* strain achieving thermotolerance in a minimal medium while maintaining homofermentative lactic acid production. A comprehensive analysis of continuous culture, transcriptomics, and comparative genomics revealed that a controlled stringent response mediated by *spoT* plays a critical role in sustaining growth during temperature upshifts. Further studies integrating proteomic and fluxomic analyses are needed to elucidate the adaptative mechanisms underlying thermal evolution in metabolically engineered strains.

## Supplementary Information

Below is the link to the electronic supplementary material.

Supplementary file1 (DOCX 62.0 KB)

## Data Availability

No datasets were generated or analysed during the current study.
