# Supplementary material for: Thermally adapted Escherichia coli keeps transcriptomic response during temperature upshift exposure
Source: Appl Microbiol Biotechnol. 2025 May 13;109(1):120. doi: 10.1007/s00253-025-13495-1 (PMC12075407; doi:10.1007/s00253-025-13495-1)
Supplement: Supplementary file 1 — Supplementary file1 (DOCX 62.0 KB) [file 253_2025_13495_MOESM1_ESM.docx]

**Applied Microbiology and Biotechnology**

Supplementary information for:

**Thermally adapted *Escherichia coli* keeps transcriptomic response during temperature upshift exposure**

Gilberto Pérez-Morales ^a^, Karla Vianey Martínez-Conde ^a^, Luis Caspeta ^a^, Enrique Merino ^b^, Miguel A. Cevallos ^c^, Guillermo Gosset ^a^, and Alfredo Martínez ^a*^

^a^ Department of Cellular Engineering and Biocatalyst. Instituto de Biotecnología, Universidad Nacional Autónoma de México. Av. Universidad 2001, Col. Chamilpa, Cuernavaca, Morelos, 62210, México.

^b^ Department of Molecular Microbiology, Instituto de Biotecnología, Universidad Nacional Autónoma de México. Av. Universidad 2001, Col. Chamilpa, Cuernavaca, Morelos, 62210, México

^c^ Program of Evolutionary Genomics, Centro de Ciencias Genómicas, Universidad Nacional Autónoma de México. Av. Universidad 2000, Col. Chamilpa, Cuernavaca, Morelos, 62210, México

^*^ Corresponding author:

AM: ORCID ID: 0000-0002-4804-6687; email: alfredo@ibt.unam.mx

Tel.: (+52) 777 3291601


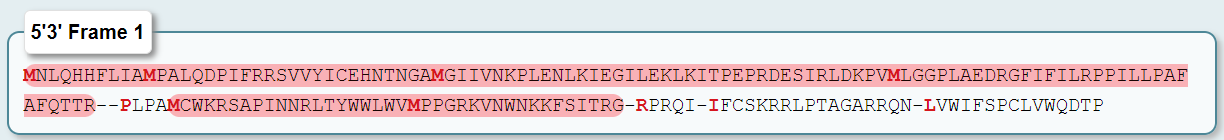


**M**NLQHHFLIA**M**PALQDPIFRRSVVYICEHNTNGA**M**GIIVNKPLENLKIEGILEKLKITPEPRDESIRLDKPV**M**LGGPLAEDRGFIFILRPPILLPAFAFQTTR--**P**LPA**M**CWKRSAPINNRLTYWWLWV**M**PPGRKVNWNKKFSITRG-**R**PRQI-**I**FCSKRRLPTAGARRQN-**L**VWIFSPCLVWQDTP

**Figure S1.** Alteration of the open reading frame by the base deletion in the *YqgF* gene. Translation of nucleotide sequence was retrieved from <https://web.expasy.org/translate/>

**Figure S2.** Growth curves of monoclonal strains isolated from the ALE end-point population. Cultures were carried out in AM1 mineral medium supplemented with 0.15 g/L of hydrolyzed protein at 45 °C.

| GO term | Biological process | JU15 strain | | | | ECL45 strain | | | |
| --- | --- | --- | --- | --- | --- | --- | --- | --- | --- |
|  |  | Early | | Late | | Early | | Late | |
|  |  | Up | Down | Up | Down | Up | Down | Up | Down |
| GO:0009987 | Cellular process | 161 | 126 | 165 | 200 | 102 | 162 | 121 | 176 |
| GO:0008152 | Metabolic process | 124 | 97 | 117 | 149 | 77 | 123 | 98 | 136 |
| GO:0050896 | Response to stimulus | 49 | 33 | 59 | 65 | 40 | 56 | 36 | 61 |
| GO:0051179 | Localization | 37 | 27 | 42 | 48 | 28 | 38 | 28 | 39 |
| GO:0065007 | Biological regulation | 38 | 31 | 32 | 35 | 29 | 34 | 34 | 44 |
| GO:0050789 | Regulation of biological process | 30 | 28 | 29 | 32 | 27 | 28 | 32 | 36 |
| GO:0051703 | Biological process involved in intraspecies interaction between organisms | 12 | 6 | 11 | 3 | 3 | 9 | 6 | 7 |
| GO:0048518 | Positive regulation of biological process | 11 | 7 | 9 | 7 | 8 | 9 | 8 | 11 |
| GO:0048519 | Negative regulation of biological process | 15 | 6 | 7 | 16 | 11 | 11 | 12 | 17 |
| GO:0023052 | Signaling | 3 | 3 | 6 | 1 | 3 | 3 | 5 | 3 |
| GO:0040011 | Locomotion | 3 | 1 | 5 | 2 | 0 | 4 | 2 | 6 |
| GO:0042592 | Homeostatic process | 6 | 4 | 5 | 5 | 2 | 2 | 3 | 7 |
| GO:0044419 | Biological process involved in interspecies interaction between organisms | 6 | 1 | 3 | 4 | 6 | 7 | 5 | 7 |
| GO:0098754 | Detoxification | 4 | 3 | 3 | 3 | 4 | 3 | 1 | 3 |
| GO:0000003 | Reproduction | 2 | 0 | 2 | 3 | 0 | 4 | 0 | 6 |
| GO:0022414 | Reproductive process | 2 | 0 | 2 | 3 | 0 | 4 | 0 | 6 |
| GO:0032502 | Developmental process | 1 | 4 | 2 | 3 | 3 | 1 | 3 | 3 |
| GO:0016032 | Viral process | 5 | 0 | 0 | 2 | 3 | 2 | 3 | 2 |
| GO:0040007 | Growth | 1 | 1 | 0 | 1 | 1 | 0 | 1 | 1 |

**Table S1.** GO terms classification by biological process of the differentially expressed genes at early and late temperature upshift exposure of parental JU15 and thermally adapted ECL45 strain. The number of differentially expressed genes in each GO term corresponds to a classification of the “Biological process” at level 2 during the stage of the temperature upshift exposure.
